# Exitrons: offering new roles to retained introns—the novel regulators of protein diversity and utility

**DOI:** 10.1093/aobpla/plae014

**Published:** 2024-03-20

**Authors:** Muhammed Shamnas v, Akanksha Singh, Anuj Kumar, Gyan Prakash Mishra, Subodh Kumar Sinha

**Affiliations:** ICAR-National Institute for Plant Biotechnology, Pusa Campus, New Delhi 110012, India; ICAR-National Institute for Plant Biotechnology, Pusa Campus, New Delhi 110012, India; Department of Botany and Plant Pathology, Lilly Hall of Life Sciences, Purdue University, West Lafayette 47906, Indiana, USA; ICAR-National Institute for Plant Biotechnology, Pusa Campus, New Delhi 110012, India; Division of Genetics, ICAR-Indian Agricultural Research Institute, Pusa Campus, New Delhi 110012, India; ICAR-National Institute for Plant Biotechnology, Pusa Campus, New Delhi 110012, India

**Keywords:** Alternative splicing, exitron, exitron retained, gene regulation

## Abstract

Exitrons are exonic introns. This subclass of intron retention alternative splicing does not contain a Pre-Terminating stop Codon. Therefore, when retained, they are always a part of a protein. Intron retention is a frequent phenomenon predominantly found in plants, which results in either the degradation of the transcripts or can serve as a stable intermediate to be processed upon induction by specific signals or the cell status. Interestingly, exitrons have coding ability and may confer additional attributes to the proteins that retain them. Therefore, exitron-containing and exitron-spliced isoforms will be a driving force for creating protein diversity in the proteome of an organism. This review establishes a basic understanding of exitron, discussing its genesis, key features, identification methods and functions. We also try to depict its other potential roles. The present review also aims to provide a fundamental background to those who found such exitronic sequences in their gene(s) and to speculate the future course of studies.

## Teaser

Exonic introns, also known as exitrons, are emerging regulatory players generated as a subclass of alternative splicing events that give rise to a new dimension to the proteome diversity of an organism that fine-tunes gene regulation at the cell level. The discovery of such sequences is expected to gain momentum in the near future due to advancements in genomics and proteomics platforms. The present review provides a fundamental background of their genesis and potential roles that might improve organisms’ suitability in a changing environment.

## Introduction

**Figure 1.**
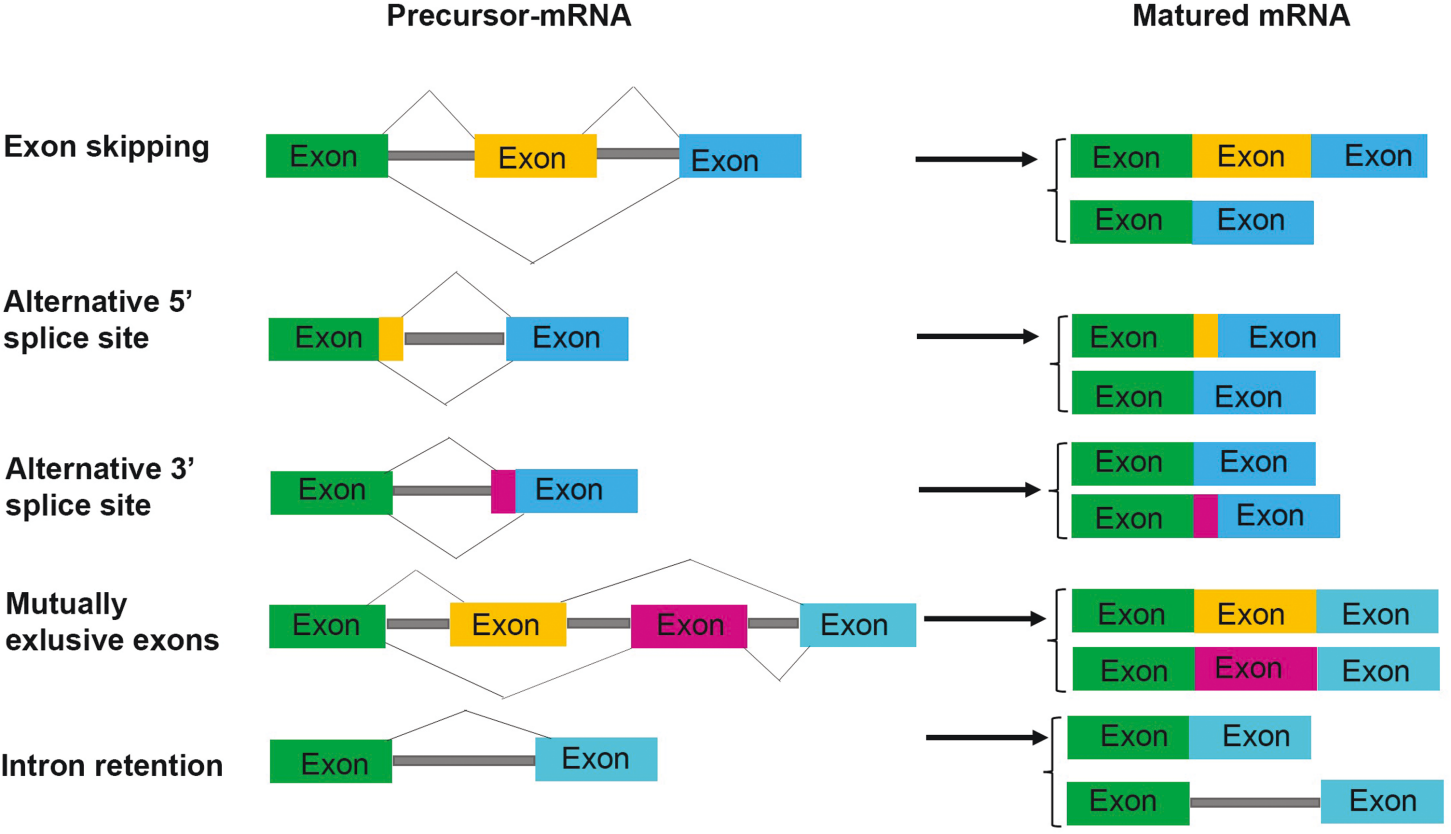
Different types of alternative splicing events.

In most eukaryotic genes, the intronic sequences interrupt the reading frame of the protein-coding sequence, that is, exons. During splicing events, the introns are removed, and consequently, exons join together to form a mature mRNA transcript. However, alternate splicing (AS) events can create multiple transcripts and proteins from a single gene by regulating the splicing events involving exons and introns (Staiger and Brown 2013). A single gene AS event can produce diverse and dynamic products whose tissue expression and abundance vary depending on developmental stages and environmental cues (Kalsotra and Cooper 2011; Staiger and Brown 2013). AS events are known to be present in about 61 % of multi-exonic genes of *Arabidopsis* (Marquez *et al.* 2012) and at least 90 % of mammalian genes (Wang *et al.* 2008), creating multiple protein isoforms with different biological functions. Major AS events include exon skipping (ES), alternative 5ʹ splice sites (A5SS), alternative 3ʹ splice sites (A3SS), mutually exclusive exons (MXEs) and intron retention (IR) (Ner-Gaon *et al.* 2004; Marquez *et al.* 2012; Braunschweig *et al.* 2013, 2014; Reddy *et al.* 2013). These AS processes, widespread in plants and humans, produce two or more mature mRNA from the same precursor-mRNA (pre-mRNA), substantial contributing to the protein diversity (Syed *et al.* 2012; Reddy *et al.* 2013) (Fig.1). ES, in which single or multiple exons are spliced out or retained along with the flanking introns, is the most common form of AS in metazoans; but is uncommon in plants (Kim *et al.* 2007). Alternative 5ʹ/3ʹ donor/acceptor sites in which two or more splice sites at one end of an exon are present alter the boundary of exons. It depends on the feasibility of using different splice sites at exon’s 5ʹ and 3ʹ ends, resulting in longer or shorter exons from the same transcript (Sugnet *et al.* 2004; Syed *et al.* 2012). Another category of AS is mutually exclusive exons (MXEs), in which only one of the two exons is retained in mature mRNA while the other exon is always spliced out (Lam *et al.* 2021). It has been shown that MXEs are found in transmembrane transporters and are involved in ion channel activity (Hatje and Kollmar. 2013; Hatje *et al.* 2017). Another type of AS is IR which is considered to be the most common form of splicing in plants and occupies 28–64 % of the total AS events, depending on the growth conditions and tissue types (Ner-Gaon *et al.* 2004; Filichkin *et al.* 2010; Kalyna *et al.*2012; Marquez *et al.* 2012; Mandadi and Scholthof 2015). IR is an AS type where introns are retained in the mature RNAs rather than getting spliced out. The IR events were initially thought to be associated with the down-regulation of gene expression through Non-sense Mediated Decay (NMD) (Ge and Porse 2014), where the premature stop codon-containing transcripts are targeted for degradation. Further IR event studies in plants show they play a significant role in regulating growth, development, physiological and stress responses (Kalyna *et al.* 2012; Syed *et al.* 2012; Drechsel *et al.* 2013; Filichkin *et al.* 2015). Originally described in plants and viruses, IR is now observed as a major form of AS in mammalian systems also (Hammarskjold 1997; Ner-Gaon *et al.* 2004; Braunschweig *et al.* 2014; Rekosh and Hammarskjold, 2018). The wide range of studies on IR that have been carried out in the mammalian system showed the importance of regulated IR mechanisms in cell development, differentiation and responses to cellular stress (Edwards *et al.* 2016; Memon *et al.* 2016; Pimentel *et al.* 2016; Ullrich and Guigo, 2020).

A subfamily of IR events where an intron constituted the internal regions of the annotated protein, they were initially reported as cryptic introns in *Arabidopsis thaliana* (Marquez *et al.* 2012). Since it is present in the internal region of protein-coding exons, the retained intron did not carry any stop codons but had core splice signals, 5ʹ and 3ʹ splice sites and branch points. Based on their dual nature (exonic and intronic), this specialized exonic–intron was named exitrons (Marquez *et al.* 2015).

Exitrons and IR possess apparent distinguishable features, leading to different fates for their transcripts. The IR transcripts are known to be retained in the nucleus as an incompletely processed transcript with forestalled translation (Boothby *et al.* 2013; Shalgi *et al.* 2014), whereas the exitron-containing transcripts are associated with polyribosome fraction and, hence, are translated. IR transcripts harbour a premature termination codon (PTC) due to the retained intron (Braunschweig *et al.* 2014), while retained exitrons-containing transcripts have no PTCs as they are protein-coding sequences. The exitron-containing transcripts are more abundant than the IR transcript (Marquez *et al.* 2012). As exitrons find their way into the complex gene regulation and proteome plasticity world, our review focuses on their genesis, exitron retention and splicing mechanism and their diverse role in plant adaptation, development and immune responses.

## Genesis of Exitrons: Unleashing the Mystery

**Figure 2.**
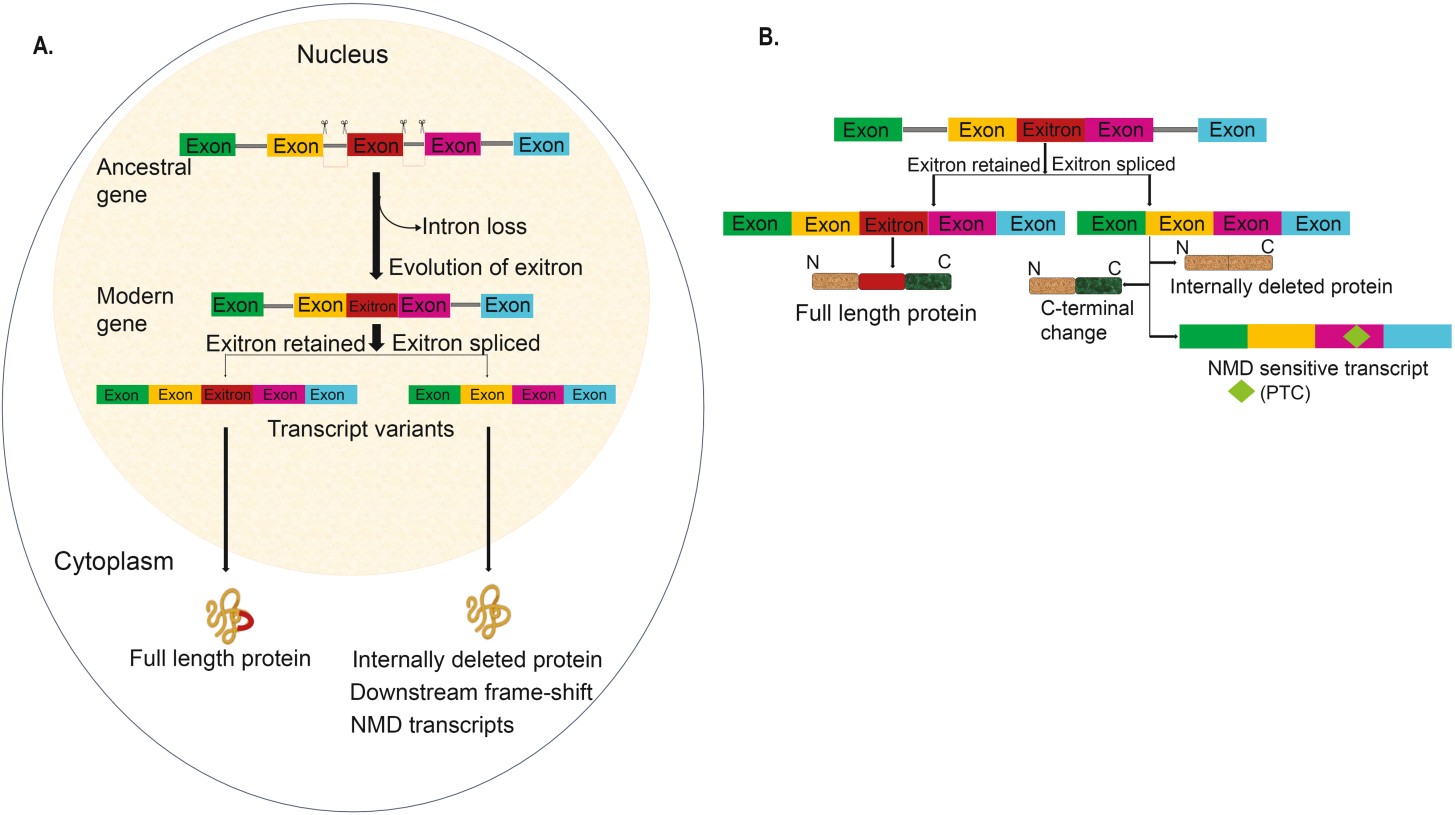
Schematic representation of genesis of exitron and exitron splicing events. (A) Exitron (shown in red) in the modern gene evolved by the loss of introns in the ancestral gene as an integral part, having features of both protein-coding exons and introns. (B) Retained exitrons produce full-length protein isoform whereas the spliced-exitron results in protein variants, namely, internally deleted protein isoform, downstream frame-shift from the splice junction causing C-terminal change in protein or producing NMD-sensitive transcripts.

Exitrons (previously called cryptic introns), having features of both introns as well as protein-coding exons, are flanked directly by exons. Therefore, exitrons have great possibilities of enhancing the protein diversity in *Arabidopsis* and humans (Marquez *et al.* 2015). The exitrons maintain their length in the multiple of three bases, thereby decreasing the chances of having a change in the reading frame. Fig. 2A shows a model depicting the generation of exitron, which regulates significant functions contributing to proteome diversity. One of the hypotheses, that is, ‘splicing memory’, has been proposed for the genesis of exitrons (Marquez *et al.* 2012, 2015), where the loss of introns from the ancestral gene causes the unusual origin of exitrons in modern genes of both plants and humans. Upon intron loss and retroposition (insertion of reverse-transcribed spliced mRNA into a new genomic position), genes have impressions of former exon borders and ‘remember’ previous exonic information. If ancestral region underwent AS, vestigial exonic splicing regulatory components that are present at exon boundaries (Reed and Maniatis 1986) can provide position-dependent details to facilitate the evolution of core splicing signals and re-establishment of modern gene structure by exitron splicing (EIS).

A conserved exitron splicing event has been observed between humans and plants (Marquez *et al.* 2015). Interestingly, retained exitrons, an internal part of a protein-coding exon, generate a longer protein as a major isoform during transcript translation. In contrast, splicing of an exitron possibly results in three types of protein variants, that is, internally deleted protein isoforms, alters the carboxyl-terminal of protein or triggers nonsense-mediated RNA decay (NMD) by introducing a PTC from the splice junction (Fig. 2B). Hence, the transcripts with both retained exitron and spliced-exitron isoforms are exported to the cytoplasm and then translated, unlike the other IR transcripts, which are found to be mostly retained in the nucleus. Hence, they affect various post-translational modifications (PTM) regulating protein function by directly targeting transcripts and increasing protein diversity and integrity. However, detailed studies about ancestral AS events, conserved splicing regulatory elements, and other aspects of EIS evolution need to be thoroughly investigated.

## Characteristic Features of Exitrons

Exitrons are highly conserved from *Arabidopsis* to human genomes. Exitrons have the following features: (i) High GC content: The exitron carries higher GC content closer to exonic GC than the conventional Intronic GC content. (ii) Weaker splice site signals: Exitrons possess weaker splice site signals than conventional introns. (iii) Absence of stop codons: In contrast to introns, exitrons do not contain stop codon(s) and thus lack premature termination of the translation event. (iv) Intron length in a multiple of three bases: Exitronic sequences are seen in a size corresponding to a multiple of three nucleotides (a rare occurrence in conventional introns) essential to maintain the ORF for a successful translation event. (v) Nuclear export: The transcripts carrying the exitron are usually transported from the nucleus to the cytoplasm for translation, while the transcripts with retained introns are incompletely processed and contained in the nucleus. (vi) Predominant transcript: The transcripts with retained introns (i.e. exitrons) are the significant isoforms compared to those with no exitron (Marquez *et al.* 2015). Exitrons (exonic introns) in the encoded protein can enrich disordered protein regions, short linear motifs and phosphorylation and ubiquitination sites, thus impacting protein function.

## Factors Affecting Exitron Splicing/Retention

Even though the factors involved in IR and AS are studied extensively, the precise mechanism of exitron retention and EIS needs to be better understood. 5ʹ and 3ʹ splice site signals and the presence of branch points are critical factors for removing introns during splicing (Wang and Burge 2008). These signals represent only a piece of information to define the introns (Lim and Burge 2001). The conditions that limit spliceosome availability, namely, down-regulation of spliceosomal components and deficient splice site recognition, affect IR events (Wong *et al.* 2013). The presence and absence of regulatory splicing cis-elements, length of the exons and introns, GC content of exon and intron, distinct DNA methylation pattern, histone modifications, nucleosome positioning over exons and introns over exon/intron boundaries are some of the factors which contribute significantly to the recognition of splice site signals and changes in splice site resulting in AS (Braunschweig *et al.* 2013; Reddy *et al.* 2013).

The transcription speed of RNA pol II may be affected by the chromatin state that may, in turn, affect the AS events (Alexander *et al.* 2010; Ullah *et al.* 2018; Zhu *et al.* 2018). The evidence for the influence of chromatin environment in the splicing process by regulating the processivity of RNA pol II and recruitment of splicing factors was proven at different times (Nojima *et al.* 2018; Jabre *et al.* 2019; Yu *et al.* 2019; Kindgren *et al.* 2020; Li *et al.* 2020; Zhu *et al.* 2020). The transcriptome (RNA-seq) and nucleosome positioning (MNase-seq) data derived from a study in *A. thaliana* elucidated the nucleosome positioning mediated control of the cold-induced alternative splicing events (Jabre *et al.* 2021). They reported that exitrons exhibited distinct nucleosome positioning patterns compared to other alternatively spliced regions. A clear difference in the nucleosome positioning pattern of exitron and other retained introns was observed, indicating their distinct regulations.

## Function of Exitrons

Exitron-retained splice variants generally manifest tissue-specific functions and play crucial roles in post-translational protein modification in plants and animals (Marquez *et al.* 2015). One of the earliest examples of an IR event that creates a novel protein isoform is the retention of intron-10 in mammals’ *NXF1* (nuclear RNA export factor 1) gene (Li *et al.* 2006). The small, nxf1, truncated protein acts as a cofactor for the nuclear export of long normal nxf1 protein, defining a novel self-gene regulation mechanism (Li *et al.* 2016).

**Figure 3.**
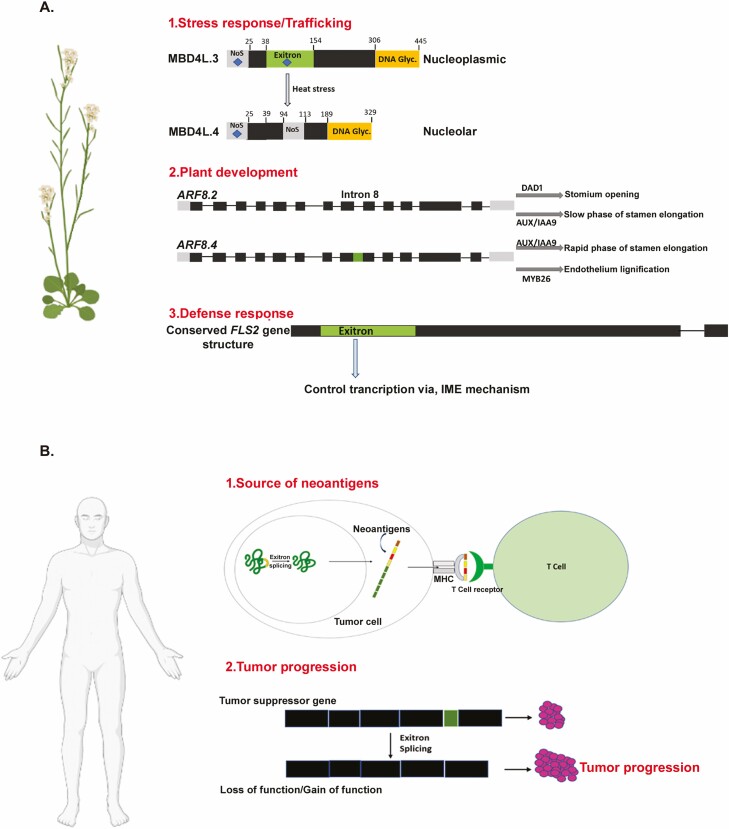
Representation of function of exitron and exitron splicing in plants and humans. Panel (A) depicts the role of exitron, and exitron and exitron splicing in plant stress response, trafficking, development and defense response. Panel (B) shows exitron acting as a source of neoantigens and their role in tumour progression.

Among the different auxin response factors reported, *ARF6* and *ARF8* play crucial roles in developing various floral organs (Nagpal *et al.* 2005). The novel splice variant of *ARF8.2*, that is, *ARF8.4*, encompassed a retained exitron (intron 8), which, upon translation, gets imported into the nucleus and regulates a developmental process that controls the growth of stamen filament and anther opening at early growth stages by activating the *MYB26* gene (Ghelli *et al.* 2018) in *Arabidopsis*. They also showed that ARF8.4 binds to specific sequences related to auxin in *AUX/IAA19* and *MYB26* promoters and further activates their transcription with enhanced efficiency than ARF8.2 (Fig. 3A). Another exitron-containing gene FLAGELLIN-SENSING 2 (*FLS2*), which codes for leucine-rich repeat receptor-like protein in plants, can sense bacterial flagellin and trigger a series of immune responses in dicot plants (Cheng *et al.* 2020). The 5ʹ splice site region of *FLS2* genes is highly conserved in dicots, and the exitron proximal to the 5ʹ end had a stimulatory role in gene expression through the intron-mediated enhancer mechanism. A protein product of the alternate spliced FLS2-1 exitron, NbFLS2-1-AT1 acts as a suppressor of ROS production induced by flagellin 22 (flg22), a potential elicitor of plant immune response suggests that one of the exitron plays a negative role in regulating the FLS2 pathway. Another study reported exitron-mediated enzyme localization in *the MBD4L* gene, which encodes DNA glycosylase. This enzyme is known to be involved in DNA repair mechanisms. Methyl-CpG-binding domain protein 4-like (MBD4L) protein is a DNA glycosylase in *Arabidopsis* which excises *in vitro* U, U-halogenated derivatives and T mis-paired to G, with preference for CpG (Ramiro-Merina *et al.* 2013). Initial studies showed that *AtMBD4L* had three alternate transcripts named *At3g07930.1*, *At3g07930.2* and *At3g07930.3* (Nota *et al.* 2015). In an attempt to amplify and clone *At3g07930.3* (*MBD4L.3*), an additional smaller-sized fragment *At3g07930.4* (*MBD4L.4*) was obtained by splicing a previously unidentified intron. Both predicted proteins contained conserved C-terminal DNA glycosylase domain (115 last amino acids) and at their N-terminus, MBD4L.3 includes two nuclear localization signals (NLS) while MBD4L.4 had one NLS (Nota *et al.* 2015). Further studies in MBD4L.3 and MBD4L.4 showed that the two isoforms had distinct localization patterns (Cecchini *et al.* 2022). MBD4L.3 is localized in the nucleoplasm, and MBD4L.4 is in the nucleolus. Interestingly, there was an increase in the nucleolar variant *MBD4L.4* under heat stress (Cecchini *et al.* 2022), showing the functional role of exitrons in trafficking under abiotic stress (Fig. 3A and Table 1).

**Table 1.** Different studies on exitrons-containing genes.

Organism	Gene	Function	Methods	References
^a^Dicots (Nine families)	*FLAGELLIN-SENSING 2* (*FLS2*)	The exitrons of *FLS2* genes control the accumulation of transcripts through an intron-mediated enhancement (IME) mechanism.	TopHat2 software, (http://ccb.jhu.edu/software/tophat/index.shtml) RT-PCR seq	Cheng *et al.* (2020)
*Arabidopsis thaliana*	*ARF8.2*	The splice variant *ARF8.2* is controlling the stomium opening (the last step in anther dehiscence) through its effect on JA biosynthesis.	qRT-PCR	Ghelli *et al.* (2018)
*Arabidopsis thaliana*	*ARF8.4*	*ARF8.4* is a splice variant that directly affects important genes involved in filament elongation and endothecium lignification.	qRT-PCR analysis	Ghelli *et al.* (2018)
*Arabidopsis thaliana*	*MBD4L*	*MBD4L* exitron can serve as a mechanism for delivering the enzyme to the nucleolus under heat stress.	RT-qPCR Nucleolar localization sequence (http://www.compbio.dundee.ac.uk/www-nod/index.jsp)	Cecchini *et al.* (2022)
*Arabidopsis thaliana*	*F Box containing protein gene* (AT2G26030)	This gene contains a 90-nt-long exitron leading to splicing the F Box binding domain in a shortened transcript.	Identified through RNA sequence dataset	Fry *et al.* (2022)
Human	*FOXO4*	*FOXO4* is known to be a tumour suppressor gene, the exon 2 mutation likely leads to loss of function due to the lack of FOXO functional domains.	RNA-Seq using ScanExitron (https://github.com/ylab-hi/ScanExitron)	Greer and Brunet (2005)
Human	*SPEN*	Splicing of this exitron in SPEN in tumour samples suggests a potential loss of its transcriptional repression function.	RNA-Seq using ScanExitron (https://github.com/ylab-hi/ScanExitron)	Ariyoshi *et al.* (2003)
Human	*TAF15, FUS, EWSR1*	These exitron-spliced genes may be involved in promoting the progression of cancer.	RNA-Seq	Wang *et al.* (2021)
Human	*MUC4, KMT2D*	*MUC4*, *KMT2D* are well-studied tumour driver genes showed both unique and complementary functions against TP53 mutation in gastric cancer.	RNA-Seq	Zhang *et al.* (2022)

^a^Rosaceae, Solanaceae, Brassicaceae, Rutaceae, Fabaceae, Crassulaceae, Fagaceae, Salicaceae and Ranunculaceae.

Exitrons, whose spliced transcripts are linked to disease pathogenesis, are also reported in humans (Sibley *et al.* 2016). The consequences of exitron splicing include indels causing in-frame changes of proteomic sequences and can introduce highly immunogenic neoantigens promoting anti-tumour immune responses (Wang *et al.* 2021). Various studies found roles of exitrons in breast cancer (Wang *et al.* 2021), prostate cancer, gastric cancer (Zhang *et al.* 2022) and other cancer types (Fig. 3B). Wang and Yang (2021), using a bioinformatic tool named ScanExitron, identified exitron splicing in 33 cancer types across 9599 tumour transcriptome data. They observed that exitron splicing affected 63 % of the coding genes of humans, and 95 % of these exitronic events were tumour specific. The exitron splicing event changed the fate of novel and known cancer genes, leading to loss or gain of function mutation, which enhances tumour progression. They identified exitron splicing-derived neo-epitopes, which can bind to MHC Class I or II and potentially be targeted for immunotherapy. An integrated protocol for the identification of exitron and exitron-derived neo-antigen using RNA-seq data using bioinformatic tools like ScanExitron and Scan Neo was developed by Wang and Yang (2021). Splicing of exitrons was observed in genes like *TAF15*, *FUS* and *EWSR1*, which may be involved in promoting cancer progression (Zhang *et al.* 2022). An exitron splicing event in the exon 2 of forkhead box protein O4 (FOXO4), implicated in regulating cell growth and cellular differentiation, upregulated significantly in tumour cells compared to normal human cells (Wang and Yang 2021).

## Methodologies for Detection

Marquez used TopHat (http://ccb.jhu.edu/software/tophat/index.shtml) alignment mapping inside the annotated protein-coding exons that led to the identification of exitrons in an intensive study of IR events in *Arabidopsis* and humans. Only those splice junctions with three reads and no mismatch in the alignment were selected to define the exitron. In this case, the exitrons carry weaker splice signals than other introns, for example, constitutive introns, alternative introns and retained introns (Marquez *et al.*, 2015). Marquez *et al.* evaluated the strength of splice sites of exitrons and introns of *A. thaliana* using position weight matrices defined by Sheth *et al.* (2006). The presence of exitron in *ARF8* and *MBD4L* genes was identified in an attempt to amplify and clone their respective reported variants (Ghelli *et al.* 2018; Cecchini *et al.* 2022). When cDNAs of different plant species were used in RT-PCR amplification of *FLS2* genes, additional bands, which arose from splicing of exitron, were observed in agarose gel. RT-PCR-seq was also further used to validate the presence of exitron in *FLS2* genes across dicots (Cheng *et al.* 2020). A bioinformatics tool, ScanExitron, was also developed by Wang and Yang (2021) for the detection and annotation of exitrons from RNA-seq data of humans. ScanExitron is a machine learning algorithm-based tool that can identify a potential exitron by analysing the transcript sequences and exon–intron boundaries, which can be further analysed for biological and functional implications. While ScanExitron analysed Illumina sequencing platform-derived short-read RNA sequence data, ScanExitronLR (Fry *et al.* 2022) utilizes long-read RNA sequences, which promises to address false-positive sequencing error often found in short-read sequences derived from repetitive regions. This tool makes use of specific annotated transcripts, that is, expectation maximization algorithm provided by LIQA (Hu *et al.* 2021) to overcome the higher sequencing errors of long reads. Outputs of ScanexitronLR can be applied to subsequent investigations of differential exitron splicing as well as exitron annotations including frameshift type, nonsense-mediated decay features and Pfam domain interruptions. Different methods adapted for identifying and validating exitron are mentioned in Table 1.

## Conclusions

**Figure 4.**
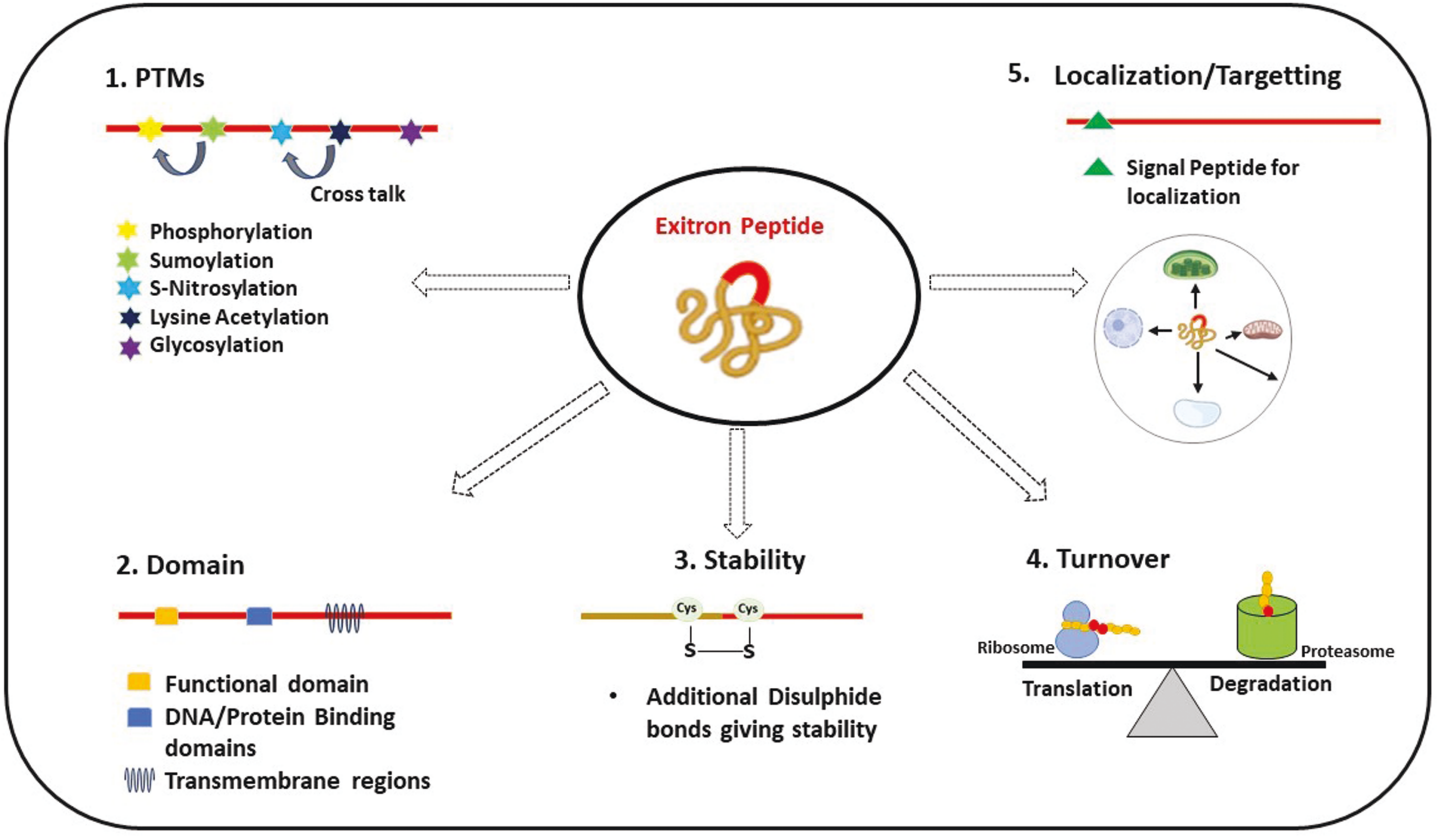
Model depicting the potential role of exitron-containing proteins. The presence of domains, phosphorylation site(s) and localization signals in the exitron peptide can give additional functional diversity to the protein. Cysteine amino acid residue could be a potential source for additional disulphide bonds enhancing the protein stability. Exitron could give a perfect balance in protein turnover.

Exitrons have only recently gained attention as an IR subclass where introns with exonic features are seen within a protein-coding conventional exon. However, with rapid advancement in RNA sequencing technologies and continued expansion of proteome and protein–protein interaction datasets, such investigation will likely lead to the discovery of many exitron-containing genes in the near future. Splicing or retaining exitrons through an AS mechanism can increase the protein diversity, thus giving diverse phenotypic changes. In plants, exitrons play key roles in their development and stress and defense response, while in humans, they are primarily associated with tumour progression. The exitron present in a gene can act as a potential site for PTM and thus have a regulatory role. Exitron can also provide suitable amino acid residues that can be a potential site for additional disulphide bonds, thus enhancing the stability of proteins. Additional domains and signal peptides within the exitron could also increase the protein diversity (Fig. 4). However, how an intron evolved into an exitron with a protein-coding feature remains a mystery. Exitrons and IR have observable distinguishing characteristics that determine a different fate for their transcripts, but a clear demarcation between the mechanisms of both events still needs to be known. Exitron splicing is also seen in annotated intronless genes, suggesting a potential source of a novel splicing mechanism. However, this emerging gene regulatory mechanism and its role in proteome complexity and phenotypic diversity need to be extensively studied in the immediate future. In this context, the integration of RNA sequencing datasets at the tissue or single cell level with information on RNA binding proteins could usher the regulation of exitron retention and splicing.

## Source of funding

The present work is financially supported by the Department of Biotechnology, Ministry of Sceince and Technology, Government of India (BT/PR42008/AGIII/103/1285/2021). The research grant was awarded to SKS.

## Data Availability

The present review does not contain any primary/secondary data as it presents a review of a recent topic (No new data generated or analysed in the present manuscript).

## References

[CIT0001] Alexander RD , Innocente SA, Barrass JD, Beggs JD. 2010. Splicing-dependent RNA polymerase pausing in yeast. Molecular Cell 40:582–593. 21095588 10.1016/j.molcel.2010.11.005 PMC3000496

[CIT0002] Ariyoshi M , Schwabe JW. 2003. A conserved structural motif reveals the essential transcriptional repression function of Spen proteins and their role in developmental signaling. Genes & Development 17:1909–1920. 12897056 10.1101/gad.266203 PMC196244

[CIT0003] Boothby TC , Zipper RS, van der Weele CM, Wolniak SM. 2013. Removal of retained introns regulates translation in the rapidly developing gametophyte of *Marsilea vestita*. Developmental Cell 24:517–529. 23434411 10.1016/j.devcel.2013.01.015

[CIT0004] Braunschweig U , Gueroussov S, Plocik AM, Graveley BR, Blencowe BJ. 2013. Dynamic integration of splicing within gene regulatory pathways. Cell 152:1252–1269. 23498935 10.1016/j.cell.2013.02.034 PMC3642998

[CIT0005] Braunschweig U , Barbosa-Morais NL, Pan Q, Nachman EN, Alipanahi B, Gonatopoulos-Pournatzis T, Frey B, Irimia M, Blencowe BJ. 2014. Widespread intron retention in mammals functionally tunes transcriptomes. Genome Research 24:1774–1786. 25258385 10.1101/gr.177790.114 PMC4216919

[CIT0006] Cecchini NM , Torres JR, López IL, Cobo S, Nota F, Alvarez ME. 2022. Alternative splicing of an exitron determines the subnuclear localization of the *Arabidopsis* DNA glycosylase MBD4L under heat stress. The Plant Journal: for Cell and Molecular Biology 110:377–388. 35061303 10.1111/tpj.15675

[CIT0007] Cheng Q , Xiao H, Xiong Q. 2020. Conserved exitrons of FLAGELLIN-SENSING 2 (FLS2) across dicot plants and their functions. Plant Science: An International Journal of Experimental Plant Biology 296:110507. 32540022 10.1016/j.plantsci.2020.110507

[CIT0008] Drechsel G , Kahles A, Kesarwani AK, Stauffer E, Behr J, Drewe P, Rätsch G, Wachter A. 2013. Nonsense-mediated decay of alternative precursor mRNA splicing variants is a major determinant of the *Arabidopsis* steady state transcriptome. The Plant Cell 25:3726–3742. 24163313 10.1105/tpc.113.115485 PMC3877825

[CIT0009] Edwards CR , Ritchie W, Wong JJ, Schmitz U, Middleton R, An X, Mohandas N, Rasko JE, Blobel GA. 2016. A dynamic intron retention program in the mammalian megakaryocyte and erythrocyte lineages. Blood 127:24–34. 10.1182/blood-2016-01-692764 PMC4850870 26962124

[CIT0010] Filichkin SA , Priest HD, Givan SA, Shen R, Bryant DW, Fox SE, Wong WK, Mockler TC. 2010. Genome-wide mapping of alternative splicing in *Arabidopsis thaliana*. Genome Research 20:45–58. 19858364 10.1101/gr.093302.109 PMC2798830

[CIT0011] Filichkin SA , Cumbie JS, Dharmawardhana P, Jaiswal P, Chang JH, Palusa SG, Reddy AS, Megraw M, Mockler TC. 2015. Environmental stresses modulate abundance and timing of alternatively spliced circadian transcripts in *Arabidopsis*. Molecular Plant 8:207–227. 25680774 10.1016/j.molp.2014.10.011

[CIT0012] Fry J , Li Y, Yang R. 2022. ScanExitronLR: characterization and quantification of exitron splicing events in long-read RNA-seq data. Bioinformatics 38:4966–4968. 36099042 10.1093/bioinformatics/btac626 PMC9620817

[CIT0013] Ge Y , Porse BT. 2014. The functional consequences of intron retention: alternative splicing coupled to NMD as a regulator of gene expression. BioEssays: News and Reviews in Molecular, Cellular and Developmental Biology 36:236–243. 24352796 10.1002/bies.201300156

[CIT0015] Ghelli R , Brunetti P, Napoli N, De Paolis A, Cecchetti V, Tsuge T, Serino G, Matsui M, Mele G, Rinaldi G, et al. 2018. A newly identified flower-specific splice variant of *AUXIN RESPONSE FACTOR8* regulates stamen elongation and endothecium lignification in arabidopsis. The Plant Cell 30:620–637. 29514943 10.1105/tpc.17.00840 PMC5894849

[CIT0016] Greer EL , Brunet A. 2005. FOXO transcription factors at the interface between longevity and tumor suppression. Oncogene 24:7410–7425. 16288288 10.1038/sj.onc.1209086

[CIT0017] Hammarskjold ML. 1997. Regulation of retroviral RNA export. Seminars in Cell & Developmental Biology 8:83–90. 15001110 10.1006/scdb.1996.0127

[CIT0018] Hatje K , Kollmar M. 2013. Expansion of the mutually exclusive spliced exome in *Drosophila*. Nature Communications 4:2460. 10.1038/ncomms3460 24025855

[CIT0019] Hatje K , Rahman RU, Vidal RO, Simm D, Hammesfahr B, Bansal V, Rajput A, Mickael ME, Sun T, Bonn S, et al. 2017. The landscape of human mutually exclusive splicing. Molecular Systems Biology 13:959. 29242366 10.15252/msb.20177728 PMC5740500

[CIT0020] Hu Y , Fang Li, Chen X, Zhong JF, Li M, Wang K. 2021. LIQA: long-read isoform quantification and analysis. Genome Biology 22:182. 34140043 10.1186/s13059-021-02399-8 PMC8212471

[CIT0021] Jabre I , Reddy ASN, Kalyna M, Chaudhary S, Khokhar W, Byrne LJ, Wilson CM, Syed NH. 2019. Does co-transcriptional regulation of alternative splicing mediate plant stress responses? Nucleic Acids Research 47:2716–2726. 30793202 10.1093/nar/gkz121 PMC6451118

[CIT0022] Jabre I , Chaudhary S, Guo W, Kalyna M, Reddy ASN, Chen W, Zhang R, Wilson C, Syed NH. 2021. Differential nucleosome occupancy modulates alternative splicing in *Arabidopsis thaliana*. The New Phytologist 229:1937–1945. 33135169 10.1111/nph.17062

[CIT0023] Kalsotra A , Cooper TA. 2011. Functional consequences of developmentally regulated alternative splicing. Nature Reviews Genetics 12:715–729. 10.1038/nrg3052 PMC3321218 21921927

[CIT0024] Kalyna M , Simpson CG, Syed NH, Lewandowska D, Marquez Y, Kusenda B, Marshall J, Fuller J, Cardle L, McNicol J, et al. 2012. Alternative splicing and nonsense-mediated decay modulate expression of important regulatory genes in *Arabidopsis*. Nucleic Acids Research 40:2454–2469. 22127866 10.1093/nar/gkr932 PMC3315328

[CIT0025] Kim E , Magen A, Ast G. 2007. Different levels of alternative splicing among eukaryotes. Nucleic Acids Research 35:125–131. 17158149 10.1093/nar/gkl924 PMC1802581

[CIT0026] Kindgren P , Ivanov M, Marquardt S. 2020. Native elongation transcript sequencing reveals temperature dependent dynamics of nascent RNAPII transcription in *Arabidopsis*. Nucleic Acids Research 48:2332–2347. 31863587 10.1093/nar/gkz1189 PMC7049701

[CIT0027] Lam SD , Babu MM, Lees J, Orengo CA. 2021. Biological impact of mutually exclusive exon switching. PLoS Computational Biology 17:e1008708. 33651795 10.1371/journal.pcbi.1008708 PMC7954323

[CIT0028] Li Y , Bor YC, Misawa Y, Xue Y, Rekosh D, Hammarskjöld ML. 2006. An intron with a constitutive transport element is retained in a Tap messenger RNA. Nature 443:234–237. 16971948 10.1038/nature05107

[CIT0029] Li Y , Bor YC, Fitzgerald MP, Lee KS, Rekosh D, Hammarskjold ML. 2016. An NXF1 mRNA with a retained intron is expressed in hippocampal and neocortical neurons and is translated into a protein that functions as an Nxf1 cofactor. Molecular Biology of the Cell 27:3903–3912. 27708137 10.1091/mbc.E16-07-0515 PMC5170612

[CIT0030] Li S , Wang Y, Zhao Y, Zhao X, Chen X, Gong Z. 2020. Global co-transcriptional splicing in arabidopsis and the correlation with splicing regulation in mature RNAs. Molecular Plant 13:266–277. 31759129 10.1016/j.molp.2019.11.003 PMC8034514

[CIT0031] Lim LP , Burge CB. 2001. A computational analysis of sequence features involved in recognition of short introns. Proceedings of the National Academy of Sciences of the United States of America 98:11193–11198. 11572975 10.1073/pnas.201407298 PMC58706

[CIT0032] Mandadi KK , Scholthof KBG. 2015. Genome-wide analysis of alternative splicing landscapes modulated during plant-virus interactions in *Brachypodium distachyon*. The Plant Cell 27:71–85. 25634987 10.1105/tpc.114.133991 PMC4330581

[CIT0033] Marquez Y , Brown JW, Simpson C, Barta A, Kalyna M. 2012. Transcriptome survey reveals increased complexity of the alternative splicing landscape in *Arabidopsis*. Genome Research 22:1184–1195. 22391557 10.1101/gr.134106.111 PMC3371709

[CIT0034] Marquez Y , Höpfler M, Ayatollahi Z, Barta A, Kalyna M. 2015. Unmasking alternative splicing inside protein-coding exons defines exitrons and their role in proteome plasticity. Genome Research 25:995–1007. 25934563 10.1101/gr.186585.114 PMC4484396

[CIT0035] Memon D , Dawson K, Smowton CS, Xing W, Dive C, Miller CJ. 2016. Hypoxia-driven splicing into noncoding isoforms regulates the DNA damage response. NPJ Genomic Medicine 1:16020. 28480052 10.1038/npjgenmed.2016.20 PMC5417364

[CIT0036] Nagpal P , Ellis CM, Weber H, Ploense SE, Barkawi LS, Guilfoyle TJ, Hagen G, Alonso JM, Cohen JD, Farmer EE, et al. 2005. Auxin response factors ARF6 and ARF8 promote jasmonic acid production and flower maturation. Development (Cambridge, England) 132:4107–4118. 16107481 10.1242/dev.01955

[CIT0037] Ner-Gaon H , Halachmi R, Savaldi-Goldstein S, Rubin E, Ophir R, Fluhr R. 2004. Intron retention is a major phenomenon in alternative splicing in *Arabidopsis*. The Plant Journal: for Cell and Molecular Biology 39:877–885. 15341630 10.1111/j.1365-313X.2004.02172.x

[CIT0038] Nojima T , Rebelo K, Gomes T, Grosso AR, Proudfoot NJ, Carmo-Fonseca M. 2018. RNA Polymerase II phosphorylated on CTD serine 5 interacts with the spliceosome during co-transcriptional splicing. Molecular Cell 72:369–379.e4. 30340024 10.1016/j.molcel.2018.09.004 PMC6201815

[CIT0039] Nota F , Cambiagno DA, Ribone P, Alvarez ME. 2015. Expression and function of AtMBD4L, the single gene encoding the nuclear DNA glycosylase MBD4L in *Arabidopsis*. Plant Science: An International Journal of Experimental Plant Biology 235:122–129. 25900572 10.1016/j.plantsci.2015.03.011

[CIT0040] Pimentel H , Parra M, Gee SL, Mohandas N, Pachter L, Conboy JG. 2016. A dynamic intron retention program enriched in RNA processing genes regulates gene expression during terminal erythropoiesis. Nucleic Acids Research 44:838–851. 26531823 10.1093/nar/gkv1168 PMC4737145

[CIT0041] Ramiro-Merina A , Ariza RR, Roldán-Arjona T. 2013. Molecular characterization of a putative plant homolog of MBD4 DNA glycosylase. DNA Repair 12:890–898. 23994068 10.1016/j.dnarep.2013.08.002

[CIT0042] Reddy AS , Marquez Y, Kalyna M, Barta A. 2013. Complexity of the alternative splicing landscape in plants. The Plant Cell 25:3657–3683. 24179125 10.1105/tpc.113.117523 PMC3877793

[CIT0043] Reed R , Maniatis T. 1986. A role for exon sequences and splice-site proximity in splice-site selection. Cell 46:681–690. 2427200 10.1016/0092-8674(86)90343-0

[CIT0044] Rekosh D , Hammarskjold ML. 2018. Intron retention in viruses and cellular genes: detention, border controls and passports. WIREs RNA 9:1470. 10.1002/wrna.1470 PMC5910242 29508942

[CIT0045] Shalgi R , Hurt JA, Lindquist S, Burge CB. 2014. Widespread inhibition of posttranscriptional splicing shapes the cellular transcriptome following heat shock. Cell Reports 7:1362–1370. 24857664 10.1016/j.celrep.2014.04.044

[CIT0046] Sheth N , Roca X, Hastings ML, Roeder T, Krainer AR, Sachidanandam R. 2006. Comprehensive splice-site analysis using comparative genomics. Nucleic Acids Research 34:3955–3967. 16914448 10.1093/nar/gkl556 PMC1557818

[CIT0047] Sibley CR , Blazquez L, Ule J. 2016. Lessons from non-canonical splicing. Nature Reviews Genetics 17:407–421. 10.1038/nrg.2016.46 PMC5154377 27240813

[CIT0048] Staiger D , Brown JW. 2013. Alternative splicing at the intersection of biological timing, development, and stress responses. The Plant Cell 25:3640–3656. 24179132 10.1105/tpc.113.113803 PMC3877812

[CIT0049] Sugnet CW , Kent WJ, Ares M Jr, Haussler D. 2004. Transcriptome and genome conservation of alternative splicing events in humans and mice. Pacific Symposium on Biocomputing. 9:66–77. 10.1142/9789812704856_0007 14992493

[CIT0050] Syed NH , Kalyna M, Marquez Y, Barta A, Brown JW. 2012. Alternative splicing in plants—coming of age. Trends in Plant Science 17:616–623. 22743067 10.1016/j.tplants.2012.06.001 PMC3466422

[CIT0051] Ullah I , Sun W, Tang L, Feng J. 2018. Roles of Smads family and alternative splicing variants of Smad4 in different cancers. Journal of Cancer 9:4018–4028. 30410607 10.7150/jca.20906 PMC6218760

[CIT0052] Ullrich S , Guigo R. 2020. Dynamic changes in intron retention are tightly associated with regulation of splicing factors and proliferative activity during B-cell development. Nucleic Acids Research 48:1327–1340. 31879760 10.1093/nar/gkz1180 PMC7026658

[CIT0053] Wang Z , Burge CB. 2008. Splicing regulation: from a parts list of regulatory elements to an integrated splicing code. RNA 14:802–813. 18369186 10.1261/rna.876308 PMC2327353

[CIT0054] Wang TY , Yang R. 2021. Integrated protocol for exitron and exitron-derived neoantigen identification using human RNA-seq data with ScanExitron and ScanNeo. STAR Protocols 2:100788. 34522901 10.1016/j.xpro.2021.100788 PMC8424586

[CIT0055] Wang ET , Sandberg R, Luo S, Khrebtukova I, Zhang L, Mayr C, Burge CB. 2008. Alternative isoform regulation in human tissue transcriptomes. Nature 456:470–476. 18978772 10.1038/nature07509 PMC2593745

[CIT0056] Wang TY , Liu Q, Ren Y, Alam SK, Wang L, Zhu Z, Hoeppner LH, Dehm SM, Cao Q, Yang R. 2021. A pan-cancer transcriptome analysis of exitron splicing identifies novel cancer driver genes and neoepitopes. Molecular Cell 81:2246–2260.e12. 33861991 10.1016/j.molcel.2021.03.028 PMC8141048

[CIT0057] Wong JJL , Ritchie W, Ebner OA, Selbach M, Wong JW, Huang Y, Emberly J, Hung NJ, Hannan KD, Buckland RB, et al. 2013. Orchestrated intron retention regulates normal granulocyte differentiation. Cell 154:583–595. 23911323 10.1016/j.cell.2013.06.052

[CIT0058] Yu X , Meng X, Liu Y, Wang X, Wang TJ, Zhang A, Li N, Qi X, Liu B, Xu ZY. 2019. The chromatin remodeler ZmCHB101 impacts alternative splicing contexts in response to osmotic stress. Plant Cell Reports 38:131–145. 30443733 10.1007/s00299-018-2354-x

[CIT0059] Zhang Y , Ye G, Yang Q, Zheng B, Zhang G, Hu Y, Yu J, Li G. 2022. Landscape of exitrons in gastric cancer. EBioMedicine 84:104272. 36137412 10.1016/j.ebiom.2022.104272 PMC9494173

[CIT0060] Zhu J , Chen Z, Yong L. 2018. Systematic profiling of alternative splicing signature reveals prognostic predictor for ovarian cancer. Gynecologic Oncology 148:368–374. 29191436 10.1016/j.ygyno.2017.11.028

[CIT0061] Zhu D , Mao F, Tian Y, Lin X, Gu L, Gu H, Qu LJ, Wu Y, Wu Z. 2020. The features and regulation of co-transcriptional splicing in *Arabidopsis*. Molecular Plant 13:278–294. 31760161 10.1016/j.molp.2019.11.004

